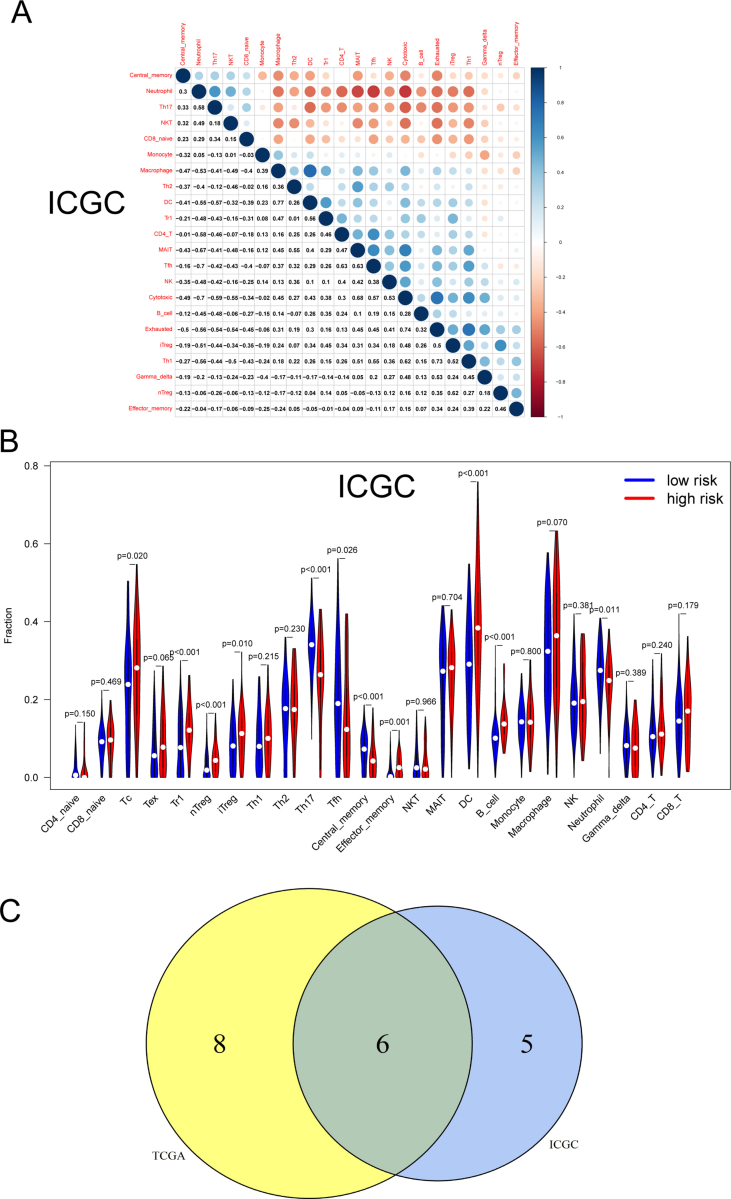# Identification of TP53 mutation-associated prognostic genes and investigation of the immune cell infiltration in patients with hepatocellular carcinoma

**DOI:** 10.1016/j.gendis.2023.03.020

**Published:** 2023-04-25

**Authors:** Qijun Yang, Lianke Gao, Yuhan Xu, Gaoquan Cao, Yingcheng He, Wenyige Zhang, Xue Zhang, Chengfeng Wu, Kaili Liao, Xiaozhong Wang

**Affiliations:** a Department of Clinical Laboratory, The Second Affiliated Hospital of Nanchang University, Nanchang, Jiangxi 330006, China; b Queen Mary College of Nanchang University, Nanchang, Jiangxi 330031, China; c Advanced Manufacturing College of Nanchang University, Nanchang, Jiangxi 330031, China; d The Forth Clinical School of Nanchang University, The South Road of Bayi Square, Nanchang, Jiangxi 330031, China; e Department of Vascular Surgery, The Second Affiliated Hospital of Nanchang University, Nanchang, Jiangxi 330006, China

Primary liver cancer, which is mainly composed of hepatocellular carcinoma (HCC), is the sixth most common type of cancer worldwide and the third most common cause of cancer mortality.^1^ The total number of mutations present in tumor specimens is called tumor mutation burden (TMB) and it is an emerging biomarker of immunotherapy response.^2^ TMB can predict clinical responses to immunotherapy such as ICI (immune checkpoint inhibitor) treatments and higher TMB is related to better survival.^3^ *TP53*, a gene encoding a tumor suppressor protein that triggers apoptosis and cell cycle arrest, is one of the most prevalent mutations in 25%–30% of HCC patients.^4^ Research shows that *TP53* mutations in HCC patients are associated with advanced tumor grade and poor prognosis.^5^ To identify the *TP53* mutation-related genes which can predict HCC patients' prognosis and explore the immune cell infiltration, we constructed a risk model based on six TP53 mutation-related genes which can accurately predict patients' prognosis. Besides, six immune cells with a similar expression pattern were identified in The Cancer Genome Atlas (TCGA) and International Cancer Genome Consortium (ICGC) databases.

The main process of this study is shown in Figure S1. Table S1 depicts the baseline characteristics of all patients retrieved from TCGA and ICGC databases. We downloaded the mutation data of 376 and 348 HCC samples from TCGA and ICGC databases respectively. These samples were then fused with corresponding clinical information according to their sample ID. There was no significant difference between samples in the TCGA dataset and those in the ICGC dataset.

Figure S2A and B shows the top 30 gene mutations of HCC according to the TCGA and ICGC databases respectively. In both databases, the frequency of asynchronous mutation in *TP53* gene is the highest, which suggests that *TP53* may play a leading role in the mutagenic mechanism of HCC. Venn diagram shows 14 genes with the highest mutation frequency of the top 30 mutant genes in the two databases (Fig. S3A). Then, we investigated the TMB differences in these 14 genes between wild type and mutation type of HCC samples, finding that the TMB of *TP53* and the other 11 genes in mutation types were significantly higher than that in wild type (Fig. S3B). Kaplan–Meier analysis was conducted on these 14 genes with patients' prognosis. Eventually, *LPR1B* and *TP53* were screened out for further research. Results showed *LPR1B* mutation was correlated with a worse prognosis while no significant difference was found between the overall survival of *TP53* mutation type and that of wild type (*TP53*, OS, *P* = 0.059; *LPR1B*, OS, *P* = 0.027) (Fig. S4A, B). Subsequently, the results of univariable Cox regression analysis suggested that TMB can serve as an independent prognostic factor (*P* < 0.01) whereas multivariate Cox regression showed that TMB cannot independently predict the prognosis of HCC patients (Fig. S4C, D). Besides, the prognostic independence of age, gender, grade, stage, and *LRP1B* mutation were also analyzed and only stage can independently predict the HCC patients' clinical outcomes (*P* < 0.01). KEGG (Kyoto Encyclopedia of Genes and Genomes) analysis showed that *LPR1B* had six representative pathways and *TP5*3 had eight (Fig. S4E, F).

We compared HCC samples with *TP53* mutation and samples without it, using the edgeR package to find DEGs (differentially expressed genes). The volcano plot shows the DEGs in *TP53* mutation-type samples (Fig. S5A). The heatmap shows the genes whose expression was obviously changed in *TP53* mutation samples compared with that in wild-type samples (Fig. S5B). We performed GO (Gene Ontology) analysis and the results revealed that these DEGs were mainly involved in the regulation of membrane potential, synaptic membrane, and channel activity. KEGG analysis indicated that these genes participated in neuroactive ligand–receptor interaction and protein digestion and absorption.

Univariable Cox regression analysis was conducted to screen out the *TP53* mutation-related genes (Table S2). LASSO regression analysis found that six genes (*SLC1A5*, *CDC20*, *SBK3*, *CTSV*, *POU3F2*, and *MYBL2*) (Table S3) were closely related to the overall survival of HCC patients, which were then used to construct our prognostic model (Fig. 1A, B). The samples in the TCGA database were used as a training set while those in the ICGC database were used as a test set. In the training set, HCC samples were further divided into high-risk and low-risk groups based on LASSO results. Figure 1C showed the risk score distribution between high- and low-risk groups. Besides, Figure 1D indicated the survival time and survival status of HCC patients in the two groups. The heatmap described the relative expression levels of six *TP53* mutation-related genes of each patient, showing that these genes were up-regulated in the high-risk group than that in the low-risk group (Fig. 1E). Survival analysis shows that the high-risk group has poorer overall survival than the low-risk group (*P* < 0.001) (Fig. 1F). In the training set, the area under the curve (AUC) showed that the 1-year, 3-year, and 5-year overall survival rates are all above 0.7, suggesting that this model is accurate in predicting the prognoses of HCC patients (Fig. 1G). As for the test set, the Kaplan–Meier survival analysis presented that the overall survival of the high-risk group was significantly lower in relation to that of the low-risk group (*P* < 0.05) (Fig. 1H). Moreover, the AUC of the 1-year overall survival rate was 0.716, which demonstrates that there was no remarkable distinction in prognosis outcomes between the two sets (Fig. 1I). In addition, both the result of multivariate Cox regression analysis and that of univariable Cox regression analysis revealed that the risk score and stage were significantly correlated with the prognosis of HCC patients (Fig. 1J, K).

**Figure 1:**
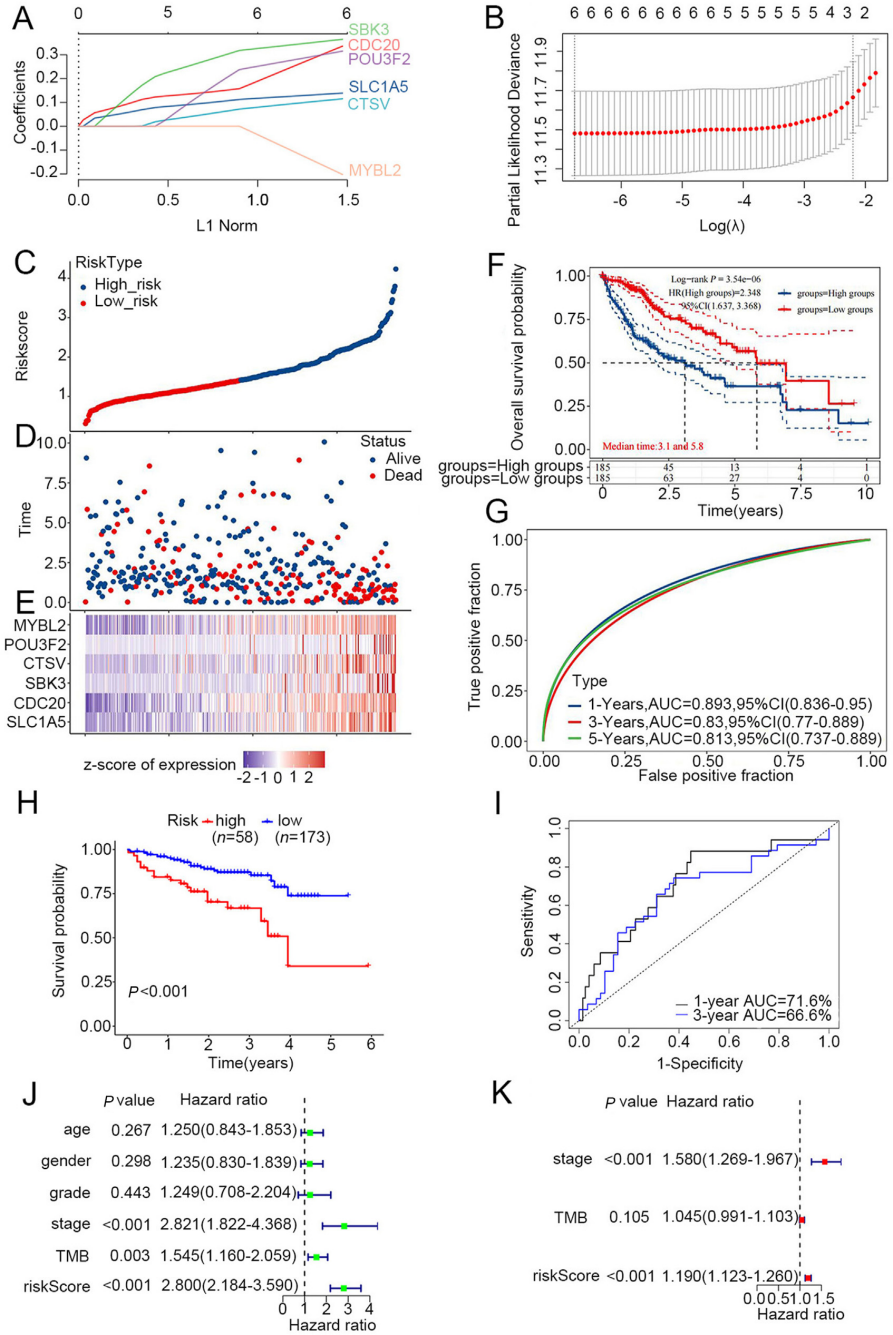
Construction of the risk model based on six *TP53* mutation-related genes. **(A, B)** The *TP53* mutation-related risk model was established via LASSO Cox regression. **(C)** The risk score distribution between the low-risk and high-risk groups of the training group. **(D)** The survival status and survival time of patients in two risk groups of the training set. **(E)** The heatmap of the expression of six genes in HCC patients' samples. **(F, H)** The nomogram for predicting patients' outcomes based on genes (*SLC1A5*, *CDC20*, *SBK3*, *CTSV*, *POU3F2*, and *MYBL2*) in the TCGA and ICGC databases. **(G, I)** The calibration curves for assessing the discrimination and accuracy of the nomogram. **(J, K)** Uni- and multivariate Cox regression analyses of the risk score of the model.

Pearson analysis demonstrated the co-expression patterns between 24 types of immune cells in the TCGA database (Fig. S6A). Additionally, the infiltration fraction of these immune cells in high- and low-risk groups in the TCGA database were compared, and a significant difference was found in the infiltration fraction of cells including Tex (exhausted T cells), nTreg (natural regulatory T cells), iTreg (induced regulatory T cells), Th1, Th17, Tem (effector memory T cells), *etc*. of the high-risk group compared with those of the low-risk group (*P* < 0.05) (Fig. S6B). The six model genes in the TCGA database were integrated into a nomogram (Fig. S7A, B). Figure S7A showed that in the TCGA database, the risk score of the model was correlated with patients' survival, with a higher score predicting poorer clinical outcomes. Figure S7B showed our model based on the TCGA database had excellent accuracy by comparing it with the calibration curve. The risk score of the nomogram model in the ICGC database is shown in Figure S7C, presenting that the risk score was negatively correlated with the survival of HCC patients. Figure S7D compared the nomogram-predicted three-year survival using the ICGC database with the actual three-year survival, elucidating the splendid accuracy of our prognostic model. Figure S8A described the co-expression patterns between 22 types of immune cells in the ICGC database. Moreover, the infiltration fraction of 24 types of immune cells was compared in the high-risk group and low-risk group, with a significant difference in Tc (cytotoxic T cells), Tr1 (T regulatory type 1 cells), nTreg, iTreg, Th17, Tfh (T follicular helper cells), central memory T cells, effector memory T cells, dendritic cells, B cells, and neutrophils (Fig. S8B). Eventually, six common cell types with a similar expression pattern were identified (Fig. S8C).

In conclusion, we established a prognostic model for HCC patients based on six *TP53* mutation-related genes which can accurately evaluate patients' prognoses and identified six immune cells with the same expression pattern in the TCGA and ICGC datasets, which may serve as biomarkers in HCC.

## Ethics declaration

This article does not contain any studies with animals performed by any of the authors. All methods are carried out in accordance with relevant guidelines and regulations.

## Author contributions

Qijun Yang designed the research and drafted the manuscript; Lianke Gao conducted the experiments; Yuhan Xu, Gaoquan Cao, Yingcheng He, Wenyige Zhang, Xue Zhang, Chengfeng Wu, and Kaili Liao did the literature search and helped draft the manuscript; Xiaozhong Wang reviewed and revised the manuscript and wrote the guidance for this work.

## Data availability

All data are available. Please contact us to access it if it is needed.

## Conflict of interests

There is no conflict of interests in this study.

## Funding

The National Natural Science Foundation of China (No. 82160405).

## Figures and Tables

**Fig. S1:**
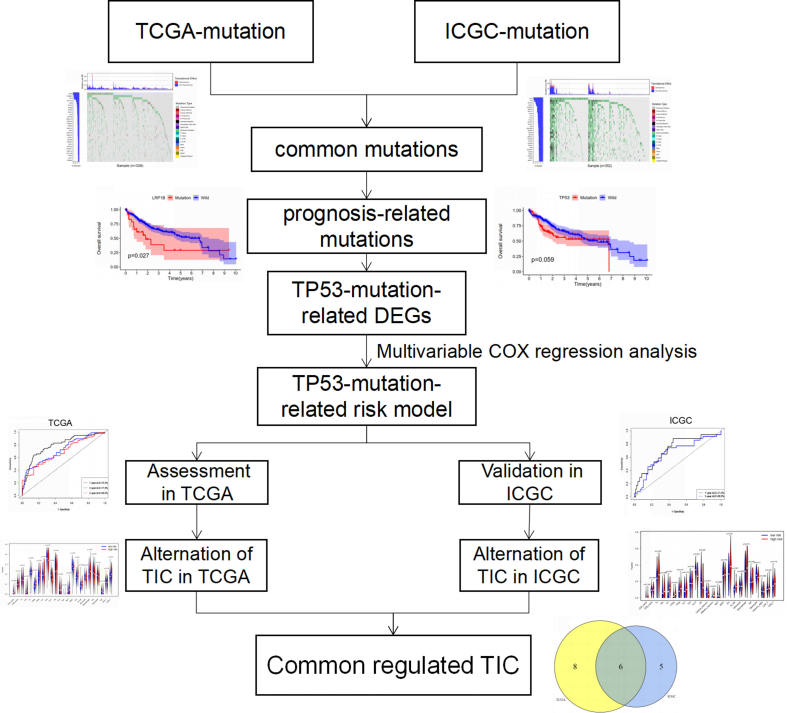


**Fig. S2:**
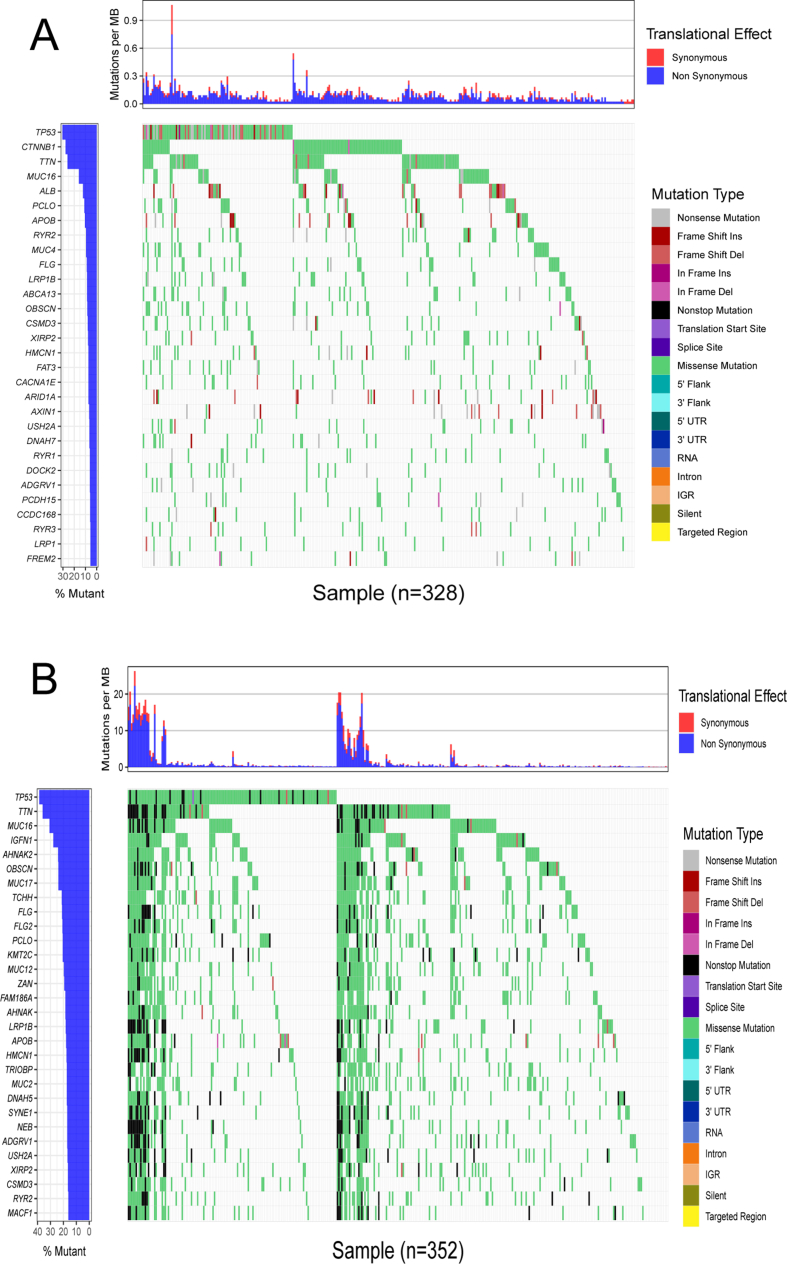


**Fig. S3:**
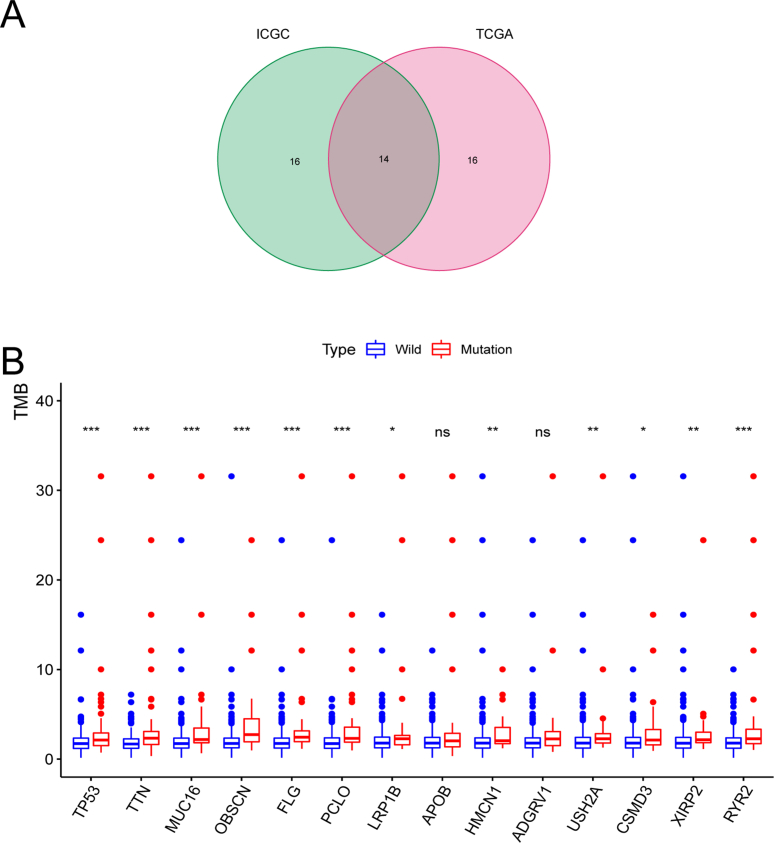


**Fig. S4:**
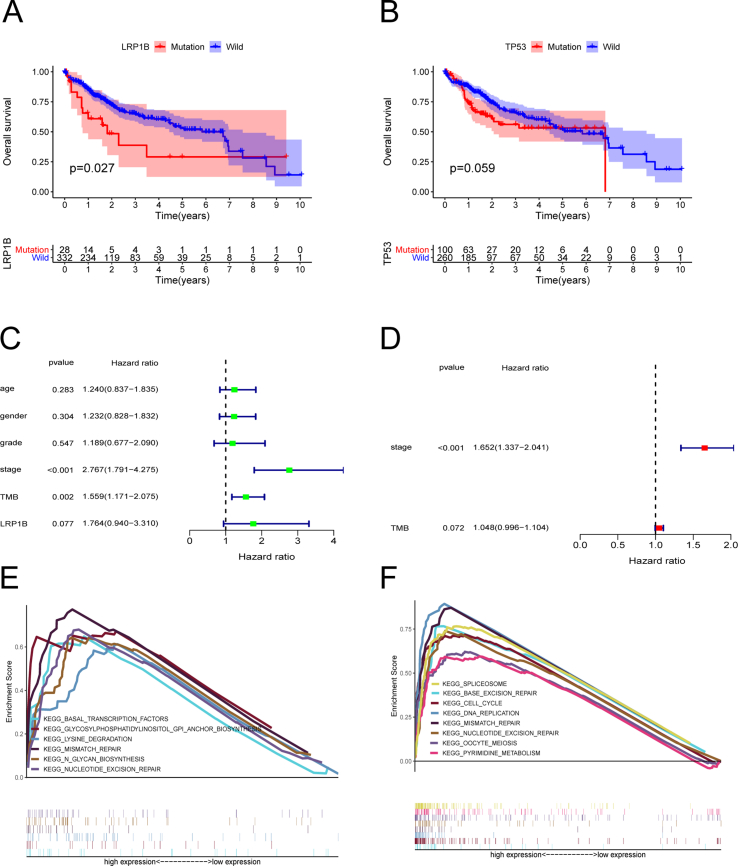


**Fig. S5:**
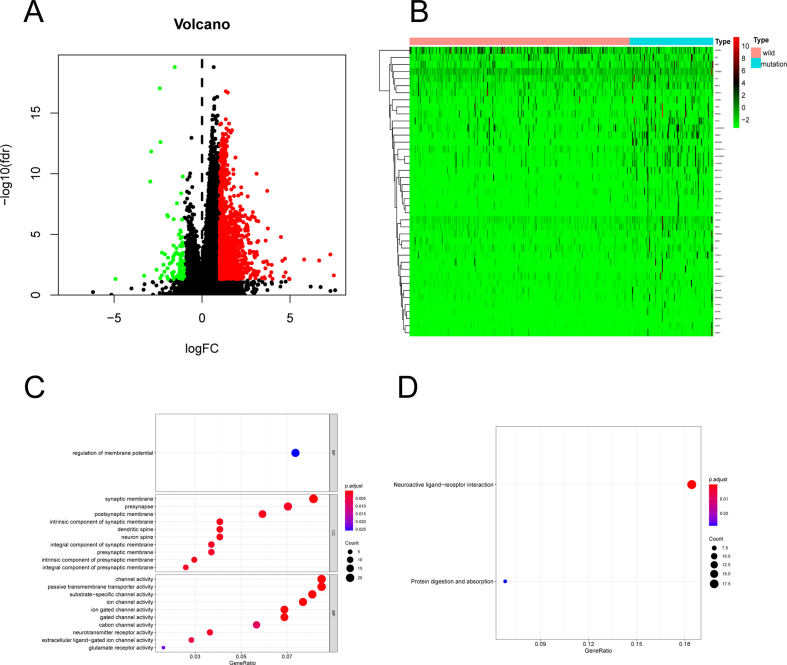


**Fig. S6:**
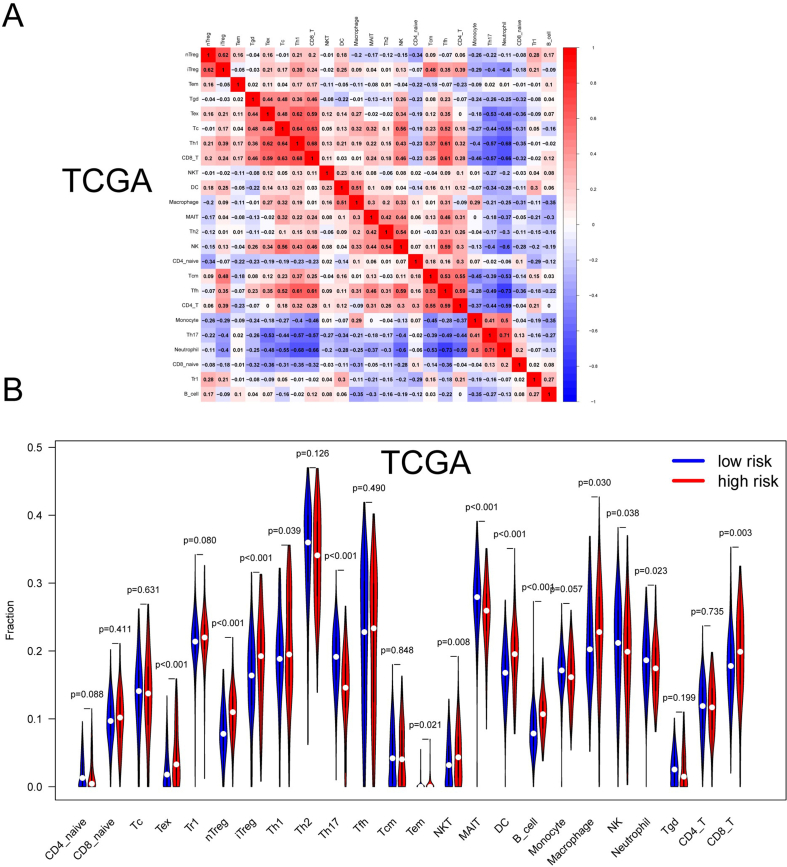


**Fig. S7:**
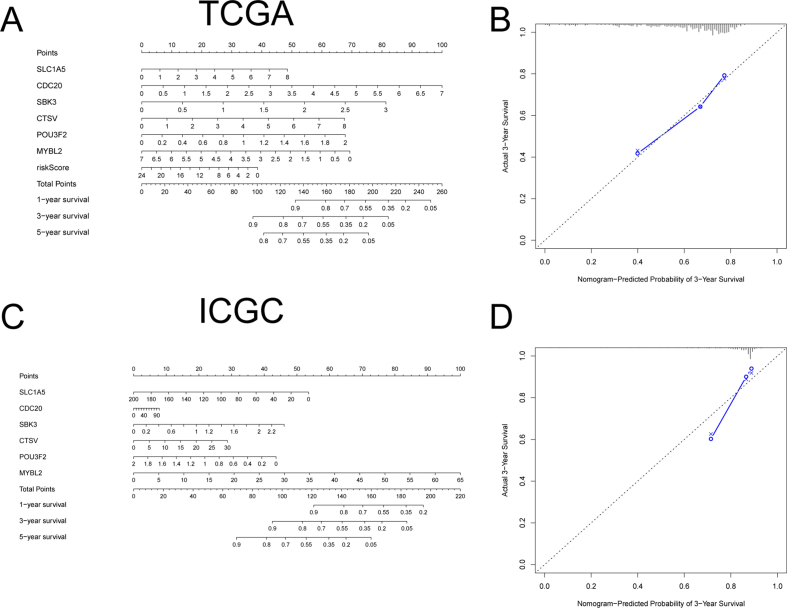


**Fig. S8:**