# Supplementary material for: Identification of TP53 mutation-associated prognostic genes and investigation of the immune cell infiltration in patients with hepatocellular carcinoma
Source: Genes Dis. 2023 Apr 25;11(2):520–3. doi: 10.1016/j.gendis.2023.03.020 (PMC10491913; doi:10.1016/j.gendis.2023.03.020)
Supplement: Multimedia component 1 [file mmc1.docx]

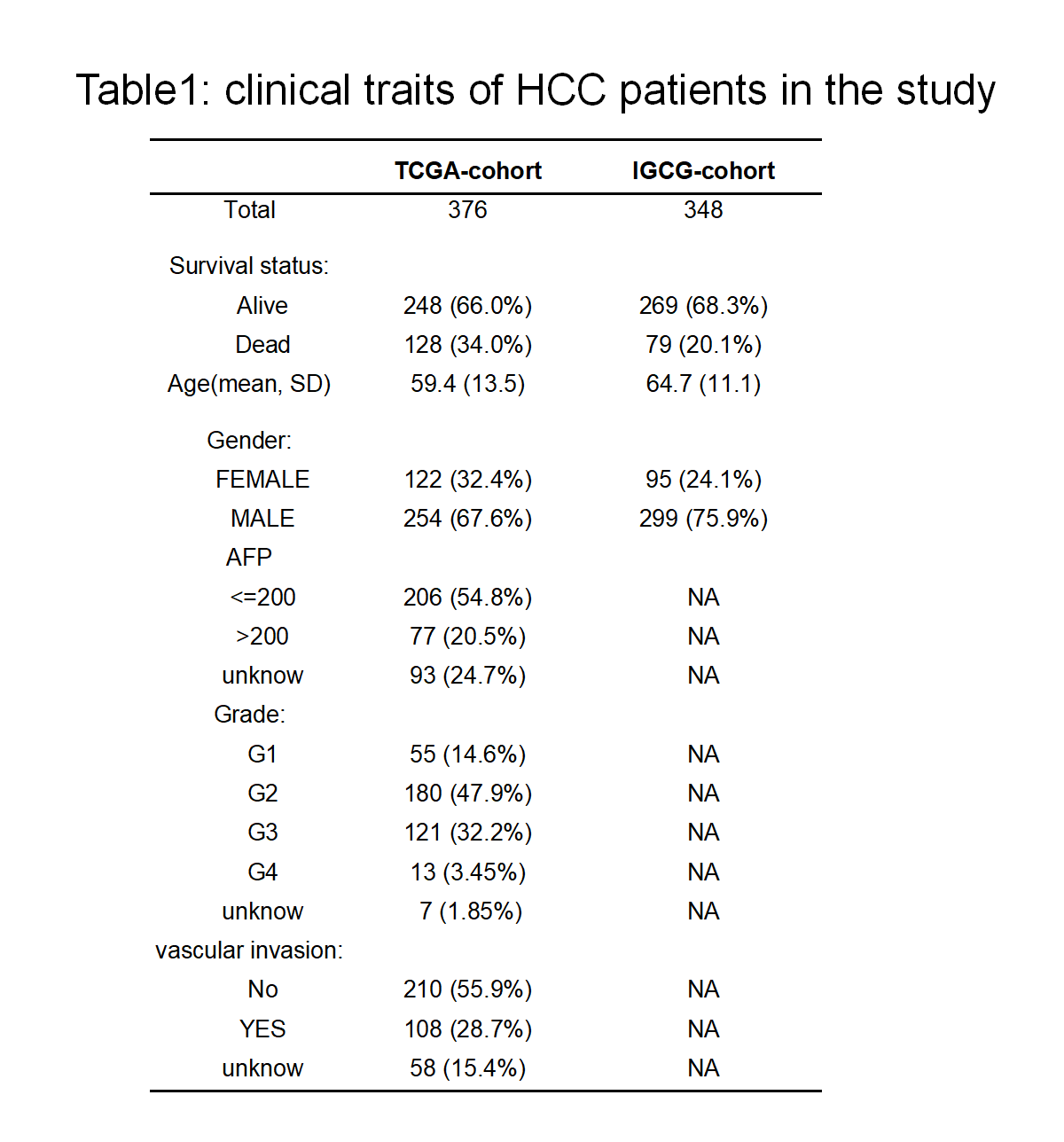
**Table S1** Clinical traits of HCC patients in the study

**Table S2** Univariable COX regression analysis of TP53-mutation-related genes

**
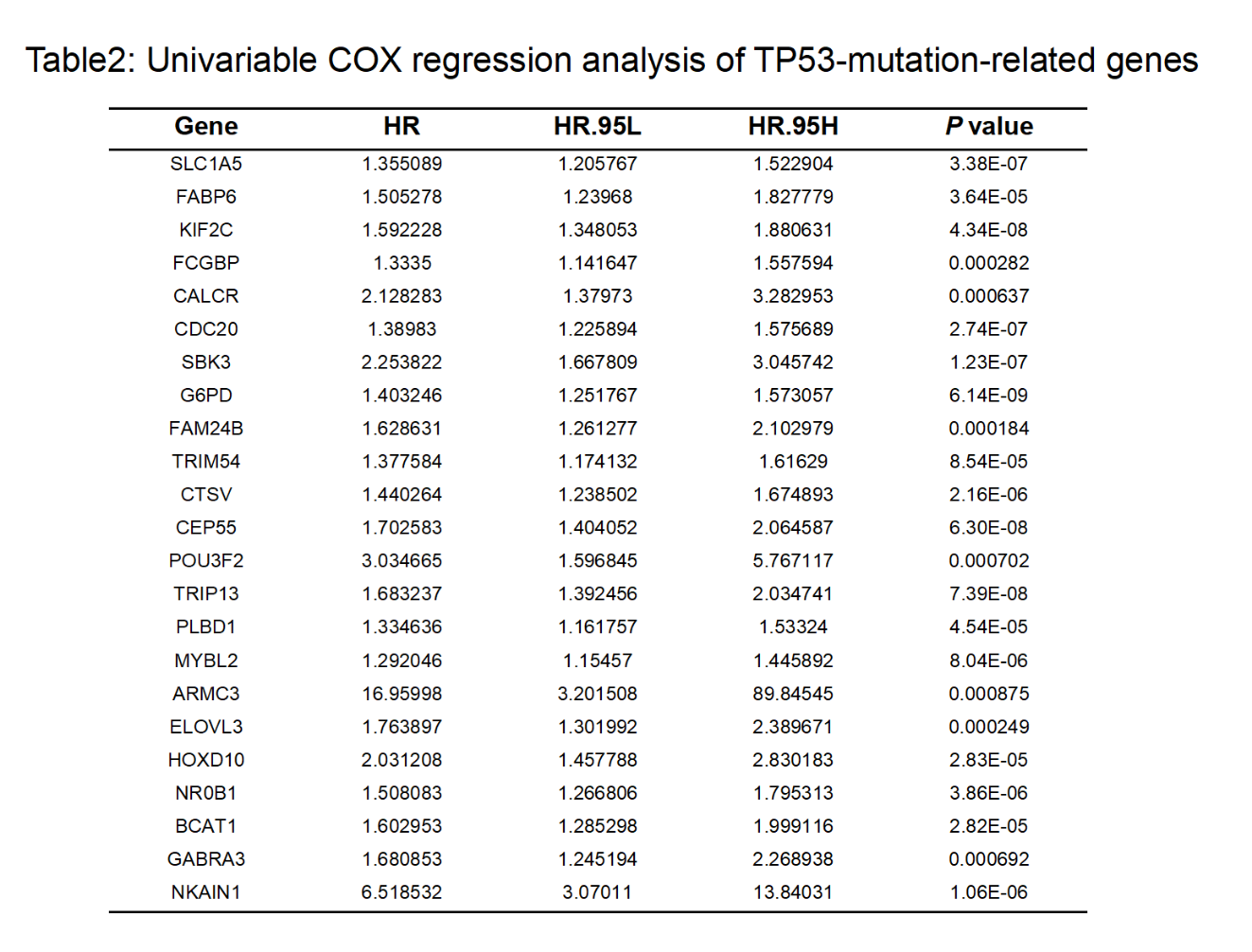
**

**
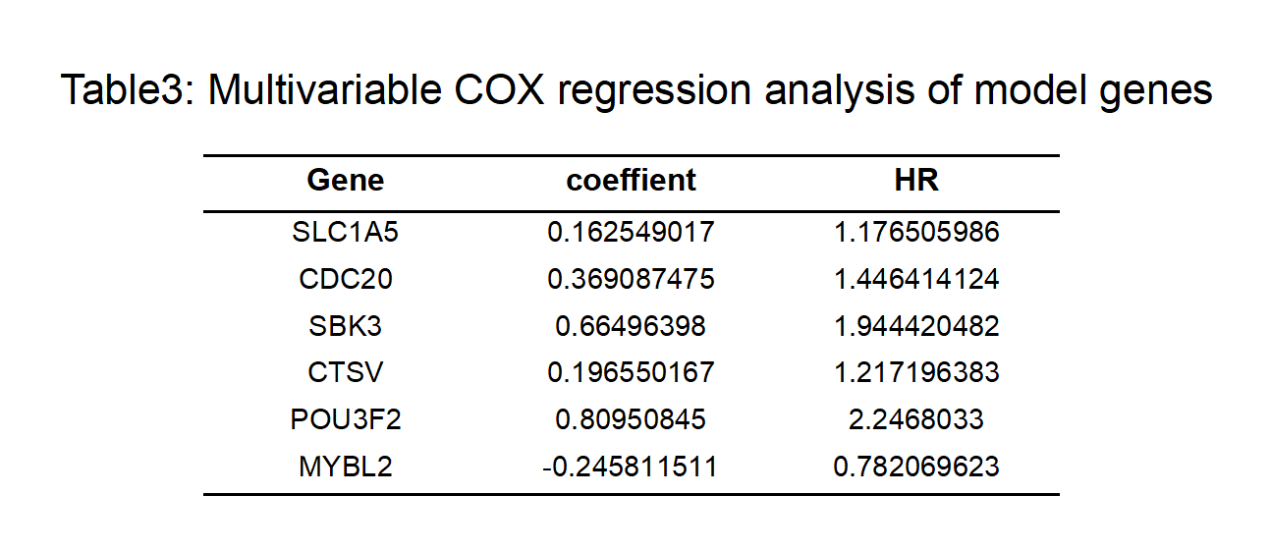
Table S3** Multivariable COX regression analysis of model genes
